# Identification and visualization of the intellectual structure and the main research lines in nanoscience and nanotechnology at the worldwide level

**DOI:** 10.1007/s11051-016-3732-3

**Published:** 2017-02-11

**Authors:** Teresa Muñoz-Écija, Benjamín Vargas-Quesada, Zaida Chinchilla-Rodríguez

**Affiliations:** 1 Department of Information and Communication, Faculty of Communication and Documentation, SCImago Research Group, University of Granada, Campus de Cartuja s/n, 18071 Granada, Spain; 2 CSIC, Institute of Public Goods and Policies (IPP), SCImago Research Group, Albasanz 26-28, 28037 Madrid, Spain

**Keywords:** Co-words, Direct citation, Intellectual structure, Research trends, Historical roots, Nanoscience & Nanotechnology, Scientometrics

## Abstract

The aim of this paper is to make manifest the intellectual and cognitive structure of nanoscience and nanotechnology (NST) by means of visualization techniques. To this end, we used data from the Web of Science (WoS), delimiting the data to the category NST during the period of 2000–2013, retrieving a total of 198,275 documents. Through direct author citation of these works, we identified their origins and the seminal papers, and through word co-occurrence extracted from the titles and abstracts, the main lines of research were identified. In view of both structures, we may affirm that NST is a young scientific discipline in constant expansion, needing time to establish its foundations but showing a strongly interdisciplinary character; its development is furthermore dependent upon knowledge from other disciplines, such as physics, chemistry, or material sciences. We believe that this information may be very useful for the NST scientific community, as it reflects a large-scale analysis of the research lines of NST and how research has changed over time in the diverse areas of NST. This study is moreover intended to offer a useful tool for the NST scientific community, revealing at a glance the main research lines and landmark papers. Finally, the methodology used in this study can be replicated in any other field of science to explore its intellectual and cognitive structure.

## Introduction

Nanoscience and nanotechnology (NST) comprise the study, design, creation, synthesis, manipulation, and application of materials, devices, and functional systems by means of the control of the material at the nanoscale, as well as the exploitation of phenomena and properties of material at the nanoscale. It is a discipline that appears in the second half of the twentieth century, when Richard P. Feynman (1960) referred to the possibilities offered by the manipulation of material at the atomic scale. The discovery of the scanning tunneling microscope (Binnig and Rohrer 1983) in the 1980s opened the gates to the development of NST, as it allowed the scientific community to obtain new materials and minute machinery.

According to the literature, only in the past decade has NST taken off on an exponential voyage due to the advancements it holds for industry, health, the environment, and national security (Huang et al. 2011). Yet, negative social and environmental implications have also come to light, as well as a lack of knowledge about the risks that many of its applications could entail (Seaton and Donaldson 2005).

Many are the works that have studied the scientific literature in NST. For example, the study run by Braun et al. (1997) established a clearly exponential growth of the NST scientific production that started in the early 1990s on the basis of a dataset with the prefix nano in the title of science and technological journal papers (4152 papers) from Science Citation Index (ISI) in the period of 1986–1995. A year later, Meyer and Persson (1998) characterized nanotechnology showing its interdisciplinary nature. Using the same approach as Braun et al. (1997), they determined that the major rate growth of nano-paper is in natural and multidisciplinary science, while the field of engineering and material and life science would be less important in view of their negative rate of growth. The growth of NST has been registered both as scientific publications and as patents. Hence, the bibliometric perspective is ideal for observing scientific activity in this discipline, with its interdisciplinary and multidisciplinary characters (Schummer 2004) and its widespread economic and social implications.

Kostoff et al. (2006b) appraised the growth of the nanotechnology research literature in the Science Citation Index, from 4552 articles in 1991 to 33,060 articles in 2004, and another study pointed out that “Global nanotechnology research article production has exhibited exponential growth for more than a decade”. They studied more than 65,000 records related to NST in the Science Citation Index/Social Science Citation Index (SCI/SSCI) in 2005 (Kostoff et al. 2007b). Porter et al. (2008) displayed the nano research publication activity from 1990 to 2006 from the Science Citation Index, finding growth in the nano research activity to be higher from the year 2000 onward. The number of papers in NST contained in WoS journals shows a steady increase since 2000 (Chen et al. 2013). Another study concludes that the number of nanotechnology journals has been growing steadily since the late 1990s (Grieneisen 2010). Such growth is also reflected in the Scopus database, where the documents published show a nearly threefold growth of NST world share output. The main growth spurt is between 2007 and 2013, a period when its world share output practically doubled (Chinchilla-Rodríguez et al. 2016a).

Beyond matters of growth, in recent years, many researchers have conducted scientometric analysis to define nanotechnology and capture its scope by using lexical queries (Porter et al. 2008; Huang et al. 2011; Grieneisen and Zhang 2011; Arora et al. 2013, 2014); to identify all disciplines that NST encompasses exhibiting the interdisciplinary of NST (Porter and Youtie 2009); to explore the interdisciplinary characteristics of NST and investigate its intellectual structure (Jo et al. 2016); to give a global overview of the infrastructure of the global nanotechnology literature through most cited authors, journal, and papers, and prolific authors, journal institutions, and countries (Kostoff et al. 2006b); to study the network of citation in nanotechnology for patents, institutions, countries, and technology fields (Li et al. 2007); to identify the core papers in NST and describe its genealogy (Kostoff et al. 2006a); to examine the cognitive structure, multidisciplinarity, and evolution of NST (Milojević 2012); or to examine the research paper and patents involved in the coming generation of active nanotechnologies to delineate phases and possible emerging research directions (Suominen et al. 2016).

In addition, bibliometric techniques have helped to study the development of certain subfields or subjects within NST, for instance carbon nanotubes (Munoz-Sandoval 2014), carbon nanostructures (Terekhov 2015), nanoparticle generation by laser ablation in liquids (Barcikowski et al. 2009), nanotoxicology (Ostrowski et al. 2009), nano-energy (Menéndez-Manjón et al. 2011; Guan and Liu 2014), or microelectromechanical systems (MENS) (Hu and Liu 2015).

Other works have applied bibliometric techniques to analyze the evolution and position of NST in the international context, so as to gauge the scientific performance of countries (Chen et al. 2013; Chinchilla-Rodríguez et al. 2016a); to compare developed and developing countries (Jafari and Zarghami 2016); to highlight activity and visibility in the global landscape of some countries, among them Russia (Terekhov 2012) or the Siberian Branch of the Russian Academy of Science (Lavrik et al. 2015), in Latin America (Invernizzi et al. 2015; Chinchilla-Rodríguez et al. 2016b), in Mexico (Lau et al. 2014), in Venezuela (López Cadenas et al. 2011), or Pakistan (Bajwa and Yaldram 2012); to evaluate the productivity, dominant research topics, and diffusion patterns in Russia, China, and India using papers from WoS and patents from US Patent and Trademark Office (USPTO) (Liu et al. 2009); to identify the risky nanomaterials that the nano-environmental, health, and safety community might examine through study of patent literature from USPTO and World Intellectual Property Organization (WIPO) and the Project on Emerging Nanotechnologies consumer product database (Leitch et al. 2012); or to identify growth trends, research topics, and the evolution in National Science Foundation (NSF) funding and commercial patenting activities at USPTO (Huang et al. 2006).

Among the array of bibliometric techniques available today, science mapping is particularly useful for analyzing and visualizing the social and intellectual structure or dynamics of scientific research fronts (Braam et al. 1991a, b; Noyons et al. 1999; Börner et al. 2003). These representations, dating back to the seventies, allow users to explore relationships involving the selected unit of analysis (Small 1973; Small and Griffith 1974; Small and Sweeney 1985; Small et al. 1985). In the 1980s, they underwent a controversial period of development and virtual brainstorming to confirm their validity (Leydesdorff 1987; Hicks 1987; Tijseen et al. 1987). This led to a slowdown in the absorption of scientific policy in the 1990s, when more user-friendly interfaces became commonplace. In the past 10 years, their use has been extended and their utility is a matter of consensus, due largely to the greater availability of data, with important expert contributions in the field of computation specializing in information visualization (Börner et al. 2003). Today’s science maps have benefited from a combination of new algorithms, the enhanced potency of calculation, and the robustness of results, as one can generate and guarantee a stable template at the worldwide level to be used as the basis for analysis (Moya-Anegón et al. 2004, 2007; Boyack et al. 2005; Leydesdorff and Rafols 2009; Rafols et al. 2010).

The development of techniques such as overlay maps (Rafols et al. 2010) makes it possible to generate such stable templates of scientific maps at the global level, for their later use to compare aspects of an organization, a field of research or the scientific output contained in databases such as the Web of Science (Thomson Corporation 2015b) or Scopus (Elsevier 2015). The cognitive structure of a scientific domain may thereby be analyzed in a geographic sense, or to perceive its evolution and the emerging lines of research that may be a priority for the economic policy of a government (Leydesdorff and Rafols 2011; Leydesdorff et al. 2015).

Such maps can thereafter serve as units of analysis regarding authors, documents, journals, or relevant term/words. Authorship is used to trace the social and intellectual structure (scientific paradigm) of a discipline, field, or specialty (Chen 1999; Garfield et al. 2003). Documents are used to visualize a knowledge domain or to assess research (Small 1999; Noyons et al. 1999). Journals can project a macro view of science or differentiation in a discipline (Leydesdorff 1987; Boyack et al. 2009).

Direct citation networks are considered the most efficient means of exploring how the scientific paradigm of a discipline is built through its own history and development (Garfield et al. 2003; Garfield 2004, 2009). Garfield points out that the most relevant nodes shown in a network constitute a landmark event in the broader development of a knowledge domain.

Although bibliometric techniques use citation analysis to study the yield of a discipline in terms of the impact or strength of influence of research efforts, using citation in the present work has nothing to do with the use (or misuse) of the journal impact factor for the promotion and financing of authors. We do not attempt to analyze the quality of research; citation is merely used to follow the flow of ideas and knowledge recorded as references made by authors when they root or support their ideas in background papers (Narin 1976; Garfield 1979). Mention of a document is a means of manifesting the relationship between a citing work and the cited work at a particular point in the development of research (Sandison 1989; Egghe and Rousseau 1990).

Our perspective with this understanding of citation focuses on identifying research topics or disciplines in cognitive networks and uncovering the intellectual structure of a discipline by the use of direct citation networks where each node represents a piece of knowledge and each link denotes the knowledge flow (Newman 2003). The analysis of citation networks, from a scientometric perspective, can help identify core literature (Potter 1988), retrace the diffusion of ideas or trace the structure of knowledge (Soper et al. 1990), or look at communication patterns, identifying the most influential authors and papers (Lawani 1980). The interpretations brought out through citation analysis applied to books and journals of a specific discipline can also testify as to its historical movement and determinant production at the national or international level (Lawani 1981).

The use of journal articles—adopted in this works as a single evaluation unit—is based on the notion that journal literature is the most formal channel of communication for scientists (Bellardo 1980) and lies at the heart of scientific and technical communication (Tomajko and Drake 1985), allowing the dissemination of information across geographical borders by scientists in any field of knowledge (Borgman 1989). In no case were journals from WoS used to quantify impact. This journal set was selected because WoS provides mature, reliable source material for mapping science (Leydesdorff et al. 2010).

Co-word analysis is a means of studying the association between the most representative terms of scientific literature, likewise lending the opportunity to obtain further information about the underlying structure and the overt tendencies of a given scientific discipline (Ding et al. 2001; Cantos-Mateos et al. 2012). Words themselves lead us to familiarity with living science and have the capacity to reflect scientific, social, and political contents that pertain to the most controversial domains or least understood emerging areas (Cantos-Mateos et al. 2014). The combination of frequency of appearance of terms, together with the techniques of spatial representation based on multidimensional scaling (MDS), provides us with information about the main research topics or those that prevail over a given period of time.

We can trace co-word analysis back to the 1980s (Callon et al. 1983; Leydesdorff 1989), when it was described as the best method to know and explain the cognitive structure of a field at the level of research specialties (Small and Griffith 1974; Braam et al. 1991a, b), moreover capable of revealing new developments of a field over time (Peters and van Raan 1993). This type of analysis was applied to polymer chemistry (Callon et al. 1991), to neural network investigation (Van Raan and Tijseen 1993), to information retrieval (Ding et al. 2001), to Information Sciences (Van Den Besselaar and Heimeriks 2006), to the links between opportunistic infections (IOs) and HIV/AIDS (Onyancha and Ocholla 2005), to the Economy (Cahlík and Jiřina 2006), to secure information (Lee 2008), Ecology (Neff and Corley 2009), to biodiversity and conservation (Liu et al. 2011), or to corporate social responsibility (Qin et al. 2016), among others.

It is assumed that NST is an attractive new scientific discipline, in which many researchers, countries, and institutions want to be involved. Its study by the use of different bibliometric, scientometric, or informetric methodologies stands as a sign of the interest that NST holds in the worldwide R&D context given the many economic benefits for developed and developing countries (Wood et al. 2003) with their variety of impending needs (Beumer 2016).

Our hypothesis stems from the premise that the bibliographic references cited in research papers are an indicator of the influence that they have on the scientific community, conditioning scientific progress and provoking new ideas and subject areas; co-word analysis furthermore marks changes in the different research lines that make up a discipline. Accordingly, our study has a triple objective. Firstly, it aims to detect the origin of and trace the evolution of the intellectual structure of NST by means of the analysis of direct citation of works. Secondly, it attempts to identify and analyze the cognitive structure over time (active research lines) by means of co-word maps to exhibit changes taking place in NST. Thirdly, the methodology described offers another perspective of NST as a discipline by means of citation and co-word analysis.

## Materials and methods

The Web of Science (WoS) was the database utilized to retrieve the set of documents on NST. Up to the time when this study was undertaken, it was the only multidisciplinary and international database containing this category, created in 2005, and composed following the Journal Citation Report (JCR) (Thomson Corporation 2015a) Science Edition 2014 by 73 journals. This source was the foundation of our search strategy. An abbreviated journal title query was subsequently designed and entered into the WoS database search engine for the version Science Citation Index Expanded 2014 (Appendix A).

Evidently, the NST category in WoS does not cover all the journals that publish articles about NST. It in-cludes only the best journals. For this reason, we decid-ed to design our search strategy using the JCR journals in the NST category, as the top journals of a discipline can be viewed as a sound tool for scientific evaluation (Garfield 1972, 1980). Although the number of NST journals has shown steady growth since the late 1990s, many articles about NST are also published in multidisciplinary science journals such as Science or Nature, or in highly specific discipline journals such as Angewandte Chemie or Applied Physics Letters (Grieneisen 2010). This is because many NST research lines in NST are not yet mature—still in the launching stage. As we needed to choose the journal set that would best represent NST as a discipline, we looked to JCR, whose journals might contain references to articles published elsewhere, that is, not exclusively in specialized NST journals.

Our search strategy used the field Publication Name, as this field is less problematic than others; it can retrieve more than 100,000 documents when combined with other fields (Arencibia-Jorge et al. 2009), yet in this case, it was limited by the time frame of study.

The period 2000–2013 was selected based on the papers of Grieneisen and Zhang (2011), who state that since 1998, nanoscale studies have grown in alarming proportions in the entire body of research. In addition, the choice of a short time period allows for the published documents to play on an equal level with the documents published previously, which may have received numerous citations; in short time periods, the documents involved are all recent and have not yet generated substantial citation (Kostoff et al. 2008). Subsequently, we chose 2013 as the last year for the sampling time frame.

The discovery of graphene in 2004, the design of new programs and software like Gaussian (2003 or 2009) or SIESTA in 2002, the use of nanowire for solar cells or as nanosensors for the detection of biological and chemical species in 2001, the characterization of gold nanoparticles in 2004, etc., these are only a few examples of core papers in NST published in the first decade of this century.

**Fig. 1:**
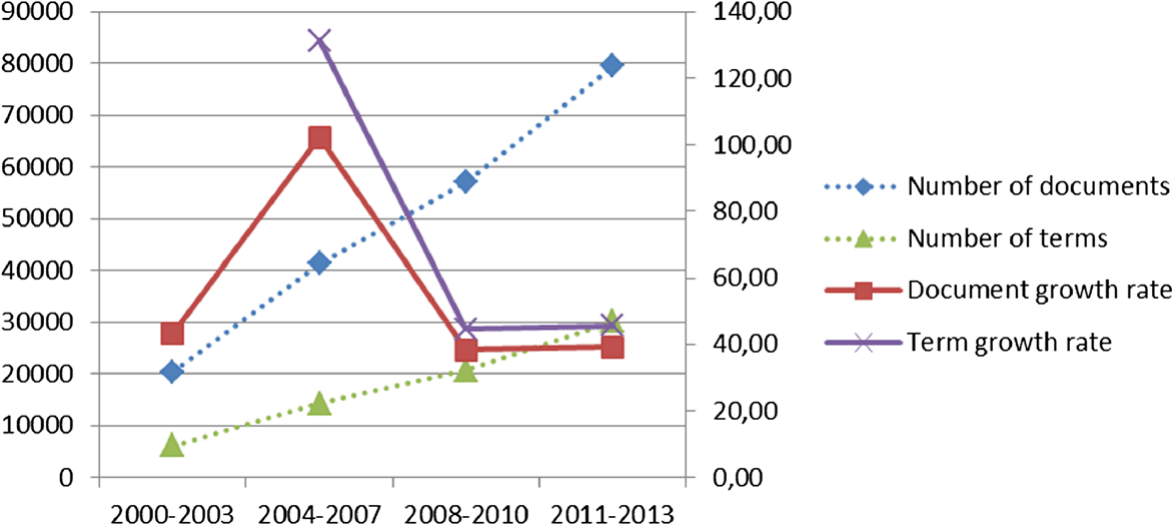
NST-retrieved documents from WoS and relevant terms

In June of 2015, we launched our search into the WoS database search engine. The number of retrieved documents (Fig. 1) from 2000 to 2013 was 198,275 and includes the following types: articles (187,949), proceedings (26,606), reviews (4066), editorial materials (3116), new items (1378), corrections (1223), letters (386), biographical items (80), meeting abstracts (70), book chapters (6), bibliographies (5), and reprints (2).

### Intellectual structure and detection of seminal papers

Citation-Assisted Background (CAB) (Kostoff and Shlesinger 2005) was used to identify seminal papers. CAB is based upon the assumption that seminal documents tend to be highly cited by the active researchers who belong to a specific scientific discipline; hence, any reference to these seminal documents will tend to be positive (Kostoff and Shlesinger 2005; Kostoff et al. 2006a). A file was generated where all downloaded documents were combined and a citation analysis was performed to identify the most cited documents of the dataset, using CitNetExplorer (Van Eck and Waltman 2014b), for visualizing and analyzing citation networks of scientific publications. This tool was used to create and visualize the intellectual network. The uncovered network featured a static structure that relied on papers as units of analysis and citations as units of measure.

The values applied to visualize information must range between 25 and 125 (White 2003). In our case, a total of 80 most-cited papers was the cutoff established, because a higher number of nodes would show too many relationships, and the viewer would perceive excessive information and an overlap of labels. This problem is known as an incomprehensible “clutter of links”.

After identifying the 80 most cited documents, we downloaded the most-cited documents which were not included in the initial dataset for one of two reasons: (a) either the documents had been published in journals that did not belong to the category of JCR NST or (b) the documents had been published before the year 2000. To download these most-cited documents from WoS, we executed different searches in the Title field, i.e., each most-cited title paper was tracked down in the Title field in WoS. This proved to be a crucial step in the process of generating the network using CitNetExplorer software. Otherwise, the nodes would have appeared as isolated nodes instead of as related nodes.

After this process, all the new downloaded papers were input into the initial dataset and a new citation analysis was performed, again using CitNetExplorer, showing the 80 most cited papers identified above and their relationships.

We should point out that downloading the most cited papers that were not included in the initial data set proved to be an essential step in our methodology, enabling us to explore the intellectual structure of NST and the relations between its nodes. If our network had only shown the most cited documents, no relationship with documents published before the year 2000 or with documents not published in JCR NST journals would have become apparent.

For the detection of the different clusters, CitNetExplorer applies an algorithm based on a variant of the modularity, by virtue of which the documents appear grouped in different clusters depending on the function of the distance in between them, so that the documents with a high degree of relatedness are found next to each other, and therefore form part of one same cluster (Van Eck and Waltman 2014a).

### Cognitive structure

Co-word maps allow us to represent the cognitive structure of a discipline, identifying the research lines that form it and detecting future lines of research by means of clustering techniques (Callon et al. 1986; He 1999).

The detection of the main research lines and their evolution using longitudinal maps or networks reveals the best consolidated lines as well as the emergent ones. Used here as the unit of analysis were the works contained in the titles and the abstracts of the set of documents retrieved (Fig. 1); their co-occurrence as units of measurement; and, as the tool for the generation and visualization of the network, VOSviewer software (Van Eck et al. 2010; Van Eck and Waltman 2015).

In an effort to control the acronyms presented by many of the terms contained in the data file, we generated a thesaurus^1^ with the most frequent acronyms. The second function of this thesaurus was to eliminate the terms of little significance contained in the documents. However, no thorough normalization of the data was carried out, which might have distorted the reality of our unit of analysis (words).

The number of words in each map increased steadily due to the rise in scientific production over the past decade. To avoid excessive noise and show only the most significant terms, a minimum occurrence threshold of 5 was established (a term was shown if it appeared at least five times). Furthermore, the VOSviewer default setting was used, meaning that 60% of the most relevant terms were displayed for each period. At any rate, a cluster should have at least ten words to be represented.

To detect the different clusters, the VOSviewer algorithm—an improved version of multidimensional scaling (MDS)—was used. This enabled us to avoid problems associated with MDS visualization such as generating circular representations or displaying the most relevant terms in the center of the representations (Van Eck and Waltman 2010).

The creation of a single map might entail some loss of information about changes in the main research lines of a discipline over time. In addition, because the number of documents per year has increased dramatically since 2000, new research trends have sprung up. With the utmost objective of representing the evolution of the cognitive structure of NST over time, without provoking a loss of structural information, we opted to make maps applying a temporary division into four sub-periods: longer periods (4 years) for the first temporal windows (2000–2003 and 2004–2007) and shorter ones for the following (3-year) periods (2008–2010 and 2011–2013) (Vargas-Quesada et al. 2010; Moya-Anegón et al. 2006). This basically resolved the problem related with the number of terms that made up each map, since the number of documents that comprised scientific output in NST in 2000–2007 is inferior to the figure for 2008–2010. Therefore, the generation of maps covering the same number of years can be said to mask reality in the displays owing to this difference in the number of terms intervening.

Each one of the circles depicted in the maps makes reference to a term, its size being proportional to the number of co-occurrences with other terms. The proximity of some terms with others indicates the association or similarity existent among them, that is, the nearer two terms are to each other, the stronger their association. To the contrary, two terms far from each other point to a low level of mutual association. The color of the circles indicates belonging to a cluster, and every cluster or grouping, with its nodes, is therefore depicted in a different color.

## Results and discussion

The average growth of NST scientific output in the time frames established for creating our maps was 55.78%. However, the period of 2004–2007 showed an outstanding growth rate of 102.17%, followed by years 2000–2003 (43.32%), 2011–2013 (39.20%), and 2008–2010 (38.41%). As for the annual growth rate, 2007 is the most remarkable year, witnessing an increase of 41.24% in NST output, followed by 2006, with 25.02%, and 2003, with a growth rate of 24.74%.

Focusing on the evolution of the number of most relevant terms for each period again signals 2004–2007 as the window of greatest growth (131.11%), after which the figures remain quite stable, from 44.66 to 45.53%.

A comparison of the average growth in the number of documents and the number of terms reveals a peak in the first periods of study, followed by a relatively stable trend in later periods.

### Intellectual structure NST—seminal papers

**Fig. 2:**
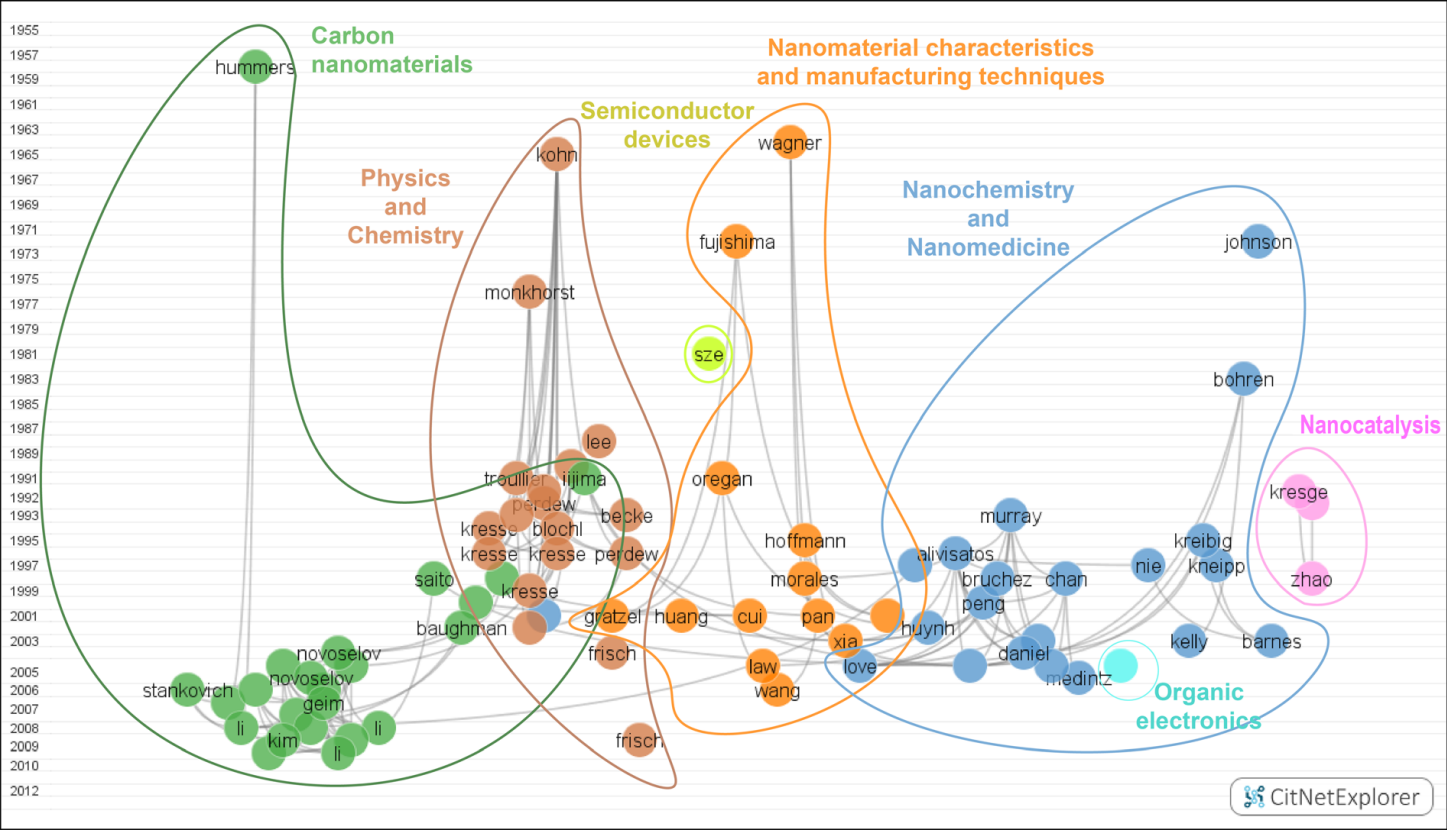
The 80 most cited document networks in NST. Available in high resolution at: http://www.ugr.es/local/benjamin/maps/Figure-2.png

Figure 2 shows the citation network with the 80 most-cited works throughout 2000–2013. The oldest of these papers detected goes back to 1958, and the most recent one is from 2009. Nodes of the citation network were cited at least 607 times from the dataset with 197,042 documents, leading to a sum total of 1,262,640 references. They appear in seven clusters that were studied selecting the Drill down option of CitNetExplorer, which offers an intuitive way of moving through the colored clusters. The maximum number of nodes in the clusters is 23 and the minimum is 1, because the nodes that form every cluster have been cited more than 607 times by the dataset.

*Blue cluster: Nanochemistry and Nanomedicine* (*21 nodes*)

**Fig. 3:**
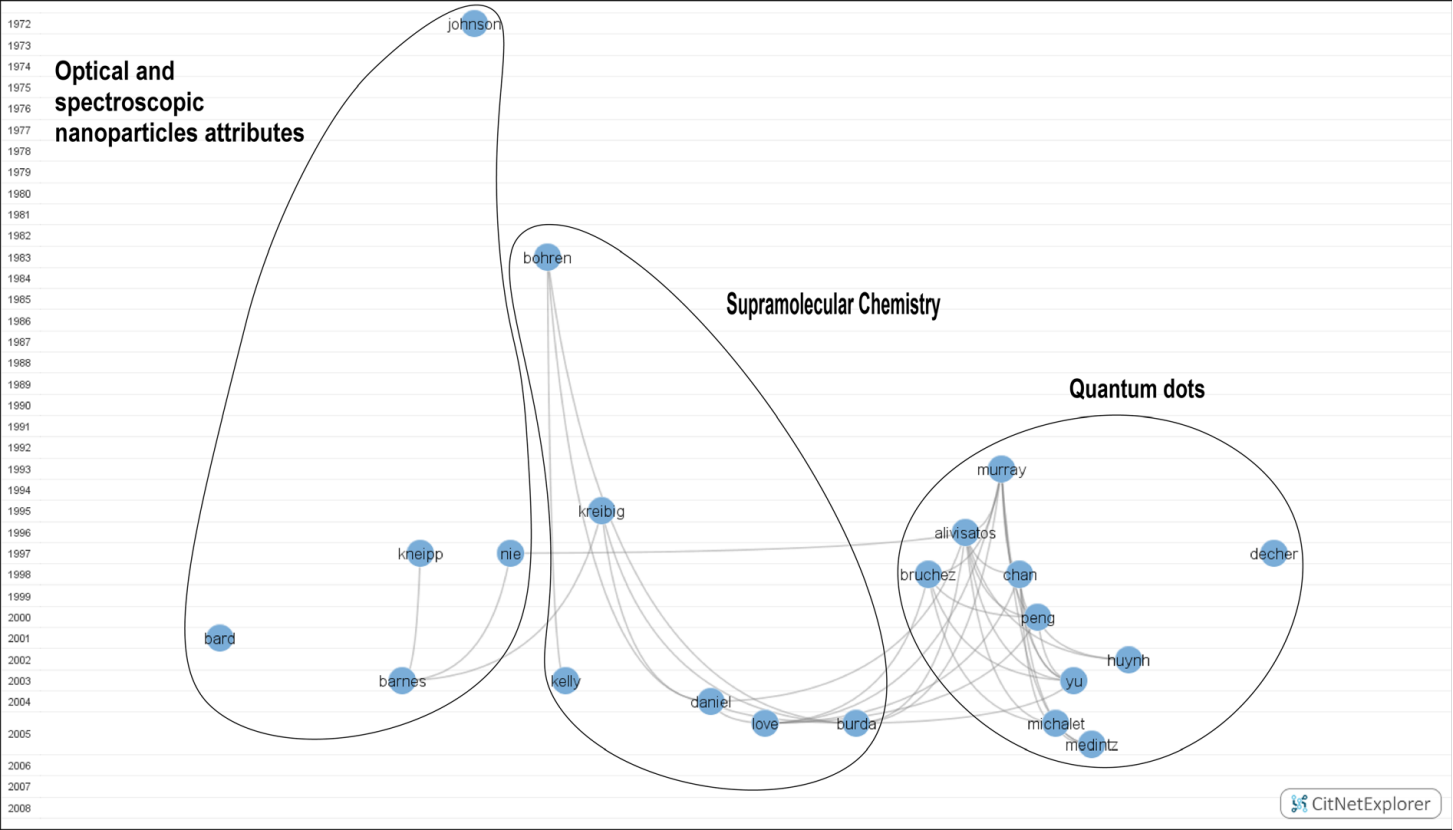
Nanochemistry and Nanomedicine. Available in high resolution at: http://www.ugr.es/local/benjamin/maps/Figure-3.png

Inside this cluster (Fig. 3), four different subgroups can be identified. To the right, we find papers by authors Murray et al. (1993), Alivisatos (1996), Decher (1997), Chan and Nie (1998), Bruchez et al. (1998), Peng et al. (2000), Huynh et al. (2002), Yu et al. (2003), Michalet et al. (2005), and Medintz et al. (2005). All of them concentrated their research activities on *Quantum dots*: composition; shape; size; and spectroscopic, electronic, and thermodynamic attributes. Alivisatos’ work is considered the most important in that area, bearing considerable influence on posterior research. In the middle are works related to *Supramolecular chemistry*. These papers were published by Bohren and Huffman (1983), Kreibig and Vollmer (1995), Kelly et al. (2003), Daniel and Astruc (2004), Love et al. (2005), and Burda et al. (2005). On the left appear papers about *Optical and spectroscopic nanoparticle attributes*. It is easy to see a major gap between the early work (Johnson and Christy 1972) and the subsequent development around 1997, when interest in this field peaked. All the areas of this cluster are seen to be associated with the research undertaken in *Nanochemistry and Nanomedicine*.

*Orange cluster: Nanomaterial characteristics and manufacturing techniques* (*13 nodes*)

**Fig. 4:**
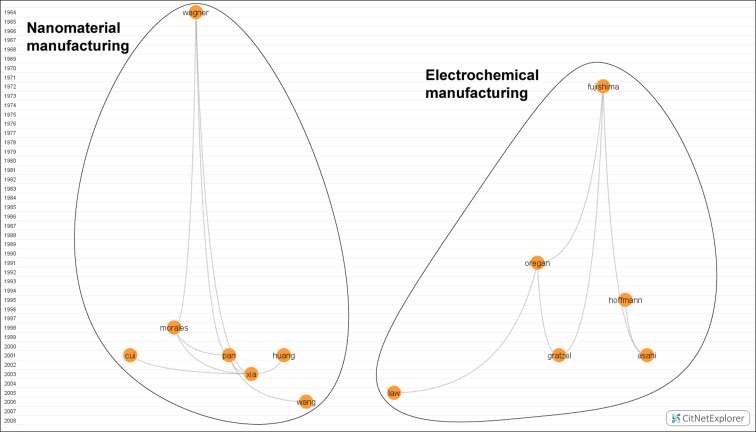
Nanomaterial characteristics and manufacturing techniques Available in high resolution at: http://www.ugr.es/local/benjamin/maps/Figure-4.png

Works of this cluster (Fig. 4) are focused on the study of bottom-up nanomaterial manufacturing techniques, although we can divide the cluster into three sections. Papers on the right explore nanomaterial manufacturing, e.g., involving nanowires or nanobelts. Wagner and Ellis’ work in 1964 was the forefather in this field (Wagner and Ellis 1964). Afterward came works by Morales and Lieber (1998), Cui et al. (2001), Pan et al. (2001), Huang et al. (2001), Xia et al. (2003), and Wang and Song (2006), the latter cited because of his review of one-dimensional nanostructures.

On the other side of the map, we find works about the manufacturing of devices to generate power energy or chemicals using electrochemistry techniques. Fujishima (1972) was the pioneer in this area, but it was not until 1991 when Oregan and Grätzel published a work that sorted out the problems suggested by Fujishima, using dye-sensitized colloidal titanium oxide to create efficient and economical solar cells (Oregan and Grätzel 1991). The papers by Hoffmann et al. (1995), Asahi et al. (2001), and Grätzel (2001) address environmental concerns through semiconductor catalysis, using solar irradiation or interior lighting to generate cheap and flexible photovoltaic cells based on nanocrystalline materials and conducting polymer films as a new generation of photoelectrochemical cells. Law’s node appears a bit separate from the others because this paper used nanowires as solar cells to further increase their efficacy (Law et al. 2005).

*Brown cluster: Physics and Chemistry* (*18 nodes*)

**Fig. 5:**
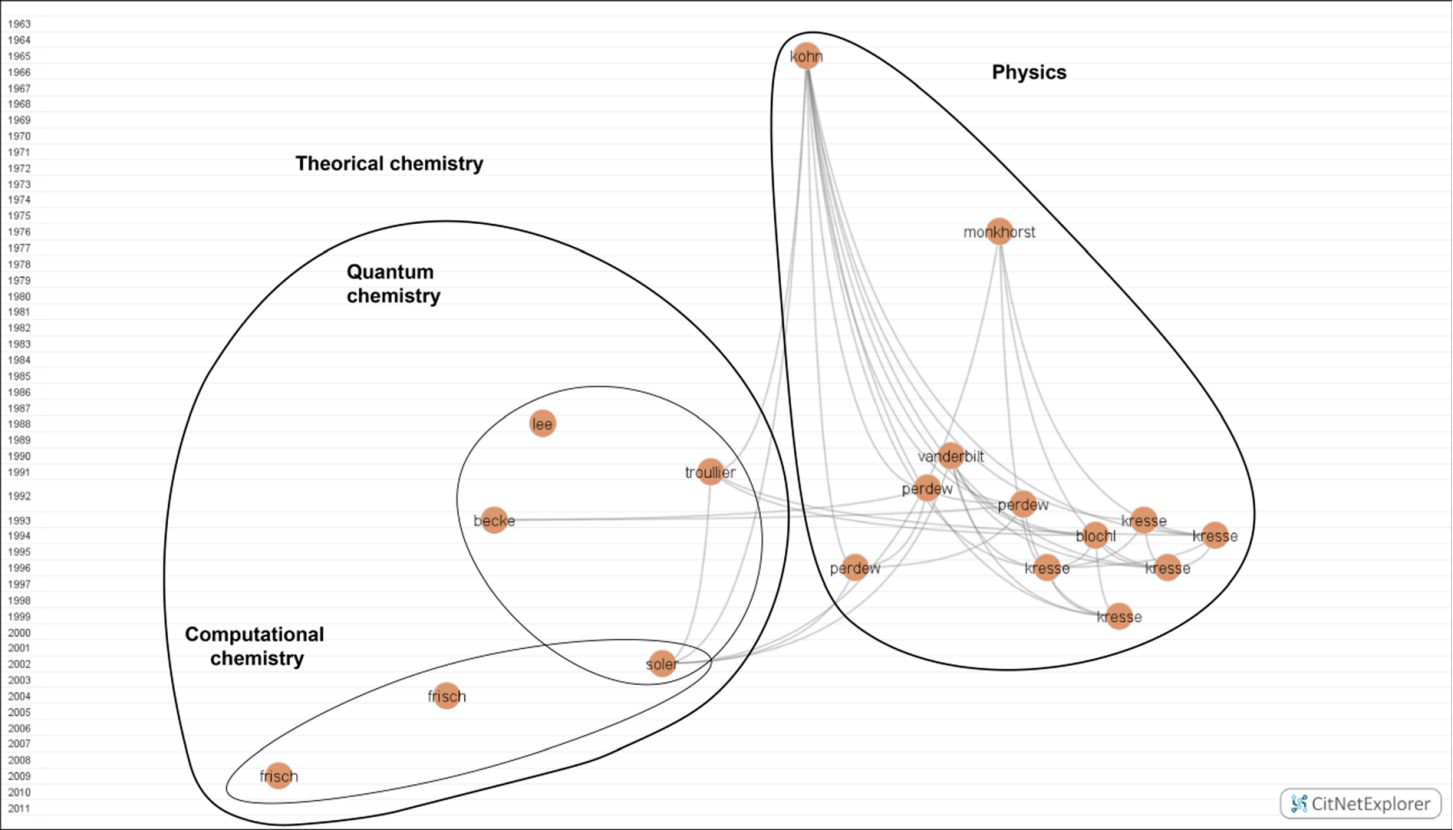
Physics and Chemistry. Available in high resolution at: http://www.ugr.es/local/benjamin/maps/Figure-5.png

Here (Fig. 5), we find the fundamental research in NST associated with Physics and Chemistry. Physics occupied the right side of the map, and Chemical theory is on the left, in turn divided into two different groups: Quantum chemistry and Computational chemistry.

On the side of Physics, deserving mention is a work from 1965 presenting equations that solved the density functional theory (DFT) (Kohn and Sham 1965), whose theorem was published the previous year. However, this theory was modified in 1990 because of its imprecision, especially for chemical applications. Another contribution to DFT is that of Perdew et al. (1996), the second most cited work in the citation network, about the exact exchange-correlation functional called generalized gradient approximation (the first did not solve Kohn and Sham’s equation). In addition, Lee et al. (1988) and Becke’s (1993) works encouraged the exchange-correlation functional.

The projector augmented wave method (PAW) was developed in order to calculate electronic structures through the pseudopotential method and linearized augmented plane-wave method (Blöchl 1994).

Kresse’s works contributed to the development of essential principles (ab-initio) of DFT, combining different methods to quickly solve Kohn and Sham’s equations (Kresse and Hafner 1993, 1994; Kresse and Furthmüller 1996a, b; Kresse and Joubert 1999). The percentage of citation of these key papers is very high, indeed comprising most of the highly cited works.

On the other side, the quantum chemistry group contains four nodes. Papers by Troullier and Soler are represented on the right. The first explored pseudopotentials, with applications to some materials through plane-wave calculations (Troullier and Martins 1991). Soler and colleagues’ work designed a program called Spanish Initiative for Electronic Simulations with Thousands of Atoms (SIESTA) that combined DFT-related methods to calculate electronic structures and ab initio molecular dynamics (Soler et al. 2002). Slightly to the left appear Lee and Becke’s works. Lee and colleagues developed a Colle-Salvetti correlation energy formula as a functional of the electron density (Lee et al. 1988), while Becke presented an improvement of the thermochemical Kohn-Sham density-functional theories with gradient corrections for exchange-correlations (Becke 1993).

The computational chemistry subgroup is located under the quantum chemistry group. The different software versions of Gaussian (03 and 09) (Frisch et al. 2004, 2009) are displayed in this group. These programs have been used in NST to run a variety of electronic structure calculations and to predict molecular properties and chemical reactions. Soler’s work also pertains to this group, as it proposed a computer program to implement SIESTA.

Most nodes here have been highly cited. Hence, it can be said that this is prestigious work presenting ideas or discoveries that proved essential for the development of NST, as it is impossible to study applied science without fundamental research.

*Green cluster: Carbon nanomaterials* (*23 nodes*)

**Fig. 6:**
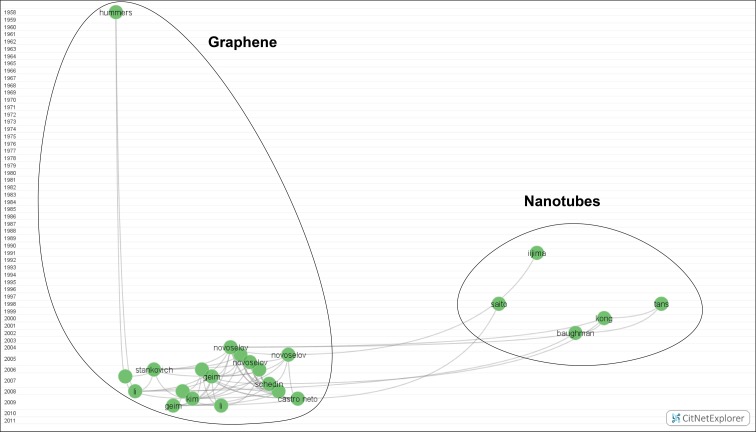
Carbon nanomaterials. Available in high resolution at: http://www.ugr.es/local/benjamin/maps/Figure-6.png

This cluster (Fig. 6) reflects a current research boom: carbon nanomaterials, such as nanotubes or graphene. The first work was published by Hummers and Offeman (1958): graphite was synthesized in order to obtain graphite oxide. Several decades later came the landmark discovery of carbon nanotubes by Iijima (1991), which revolutionized NST and inspired prolific research by Tans et al. (1998), Kong et al. (2000), Baughman et al. (2002), and so on. Nanotubes led to the development of graphene in Novoselov et al. (2004). Nowadays, many basic and applied research efforts are focused on graphene—how to synthesize this nanomaterial and how to handle it without breaking or modifying it or finding new applications according to its electronic, optic, thermal, and mechanic characteristics. Hence, as the map shows, all nodes below the graphene node have dealt with this material.

A glance at the green cluster shows that many nodes have been cited in a short period of time. In other words, work with graphene tends to be highly cited. This new research line shows great promise for the future.

*Pink cluster: Nanocatalysis* (*3 nodes*)

Works in this cluster (Fig. 2) involve the synthesis of nanoporous materials and the application of mesoporous (pore size to 2 from 5 nm) materials in the synthesis of chemical products, known as nanocatalysis (Kresge et al. 1992; Beck et al. 1992; Zhao et al. 1998).

*Light blue cluster*: *Organic electronics* (*1 node*)

Research on producing photovoltaic power using polymer devices is displayed in this cluster (Fig. 2). In particular, Li and colleagues’ work studied photovoltaic polymer cells whose electric characteristics (conductivity) lend them to further applications (Li et al. 2005).

*Yellow cluster*: *Semiconductor devices* (*1 node*)

There is only one node is this cluster: Sze (1981). This work is held to be a main reference in the field of *semiconductor devices* (Fig. 2).

### Cognitive structure—research patterns and emerging trends

This section shows the evolution of NST research from 2000 to 2013 by means of science maps generated with VOSviewer. Tables 1 and 2 make it easy to schematically view the clusters identified, using the VOSviewer algorithm, in the different periods and their relationships with the rest of the clusters.

**Table 1:**
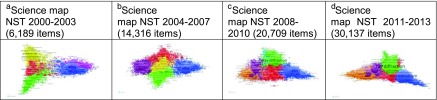
Science maps NST

^a^Available in high resolution at: http://www.ugr.es/local/benjamin/maps/N&N-2000-2003.png

^b^Available in high resolution at: http://www.ugr.es/local/benjamin/maps/N&N-2004-2007.png

^c^Available in high resolution at: http://www.ugr.es/local/benjamin/maps/N&N-2008-2010.png

^d^Available in high resolution at: http://www.ugr.es/local/benjamin/maps/N&N-2011-2013.png

**Table 2:** Identified clusters between 2000 and 2013

2000–2003	2004–2007	2008–2010	2011–2013
Microelectronics engineering and top-down processes	Microelectronics engineering and top-down processes	Microelectronics engineering and top-down processes	Microelectronics engineering and top-down processes + Organic electronics
Synthesis of nanomaterials and bottom-up processes + Biotechnology and Biomedicine	Synthesis of nanomaterials and bottom-up processes	Synthesis of nanomaterials and bottom-up processes + Optics and Electronics	Synthesis of nanomaterials and bottom-up processes + Optics and Electronics
Mechanical characteristics of materials	Mechanical characteristics of materials + Physical characteristics of materials	Mechanical characteristics of materials + Physical characteristics of materials	Mechanical characteristics of materials + Physical characteristics of materials
Optics and Electronics	Optics and Electronics	Biotechnology and Biomedicine: Biosensing	Biotechnology and Biomedicine: Biosensing
Physical characteristics of materials	Biotechnology and Biomedicine	Biotechnology and Biomedicine: Therapeutic biomedicine	Biotechnology and Biomedicine: Therapeutic biomedicine
		Organic electronics	

Tables 3, 4, 5, and 6 present the 15 terms with the highest score (according to VOSviewer) for each cluster and for each period studied. None of these 15 terms is repeated in other periods. Yet, if we increase the cutoff to 30 or even to 50 terms, there are repetitions—even though the order of occurrence varies. This is an indication that the evolution of NST research is quite rapid, and the research lines tend to change as new advances are made (Tables 7 and 8).

**Table 3:** Most relevant terms of Optics and Electronics cluster

2000–2003	Score	2004–2007	Score	2008–2010 (joined SNBUP)	Score	2011–2013 (joined SNBUP)	Score
External voltage	7.00	Nanoscale morphology	16.58	Electric vehicle	11.16	Semiconductor photocatalysis	13.09
Lasing action	5.59	Charge collection	15.00	Pulverization	9.64	MoSe2	12.83
Individual single walled carbon nanotube	5.32	Improved performance	11.29	Graphite anode	9.30	mAh g1	9.79
Quantum rod	4.55	Polymer solar cell	10.49	Battery electrode	9.16	Visible light photocatalytic hydrogen	8.98
Logic function	4.29	Bulk heterojunction solar cell	10.04	Rechargeable lithium battery	8.53	Advanced electrode material	8.89
Kilowatts per square centimeter	3.60	Donor acceptor interface	9.61	Charge capacity	8.13	Robust adhesion	8.42
Electrical transport measurement	3.55	Charge generation	9.54	Energy storage device	7.48	Asymmetric supercapacitor	8.35
Transport study	3.39	Biological labeling	9.12	Cycle life	7.41	Flexible energy storage device	8.21
Low temperature growth	3.31	Plastic solar cell	8.30	Fascinating property	7.39	Superior cyclability	8.21
Modules for experiments in stellar astrophysics	3.06	Graphene nanoribbon	8.05	Supercapacitor electrode material	7.09	High energy lithium ion battery	8.11
Density of states	3.02	Poly3 hexylthiophene butyric acid methyl ester	7.95	High charge storage capacity	6.81	Capacitive energy storage	7.88
Laser emission	3.02	Semiconducting polymer	7.94	Electrochemical energy storage	6.80	New electrode material	7.87
CdSe quantum dot	2.80	Solid state lighting	7.69	Hydrocarbon fuel	6.77	Excellent mechanical strength	7.70
Dimensional plasmon	2.77	Overall conversion efficiency	7.10	Capacity fading	6.75	High performance libs	7.66
Miniband transport	2.77	Multiple exciton generation	7.06	Gradual reduction	6.64	Nanostructured electrode material	7.27

**Table 4:** Most relevant terms of Synthesis of nanomaterials and bottom-up processes cluster

2000–2003 (Joined Biotechnology and Biomedicine)	Score	2004–2007	Score	2008–2010 (Joined Optics and Electronics)	Score	2011–2013 (Joined Optics and Electronics)	Score
ZnO nanowire	20.81	Nanometer wall thickness	11.13	Electric vehicle	11.16	Semiconductor photocatalysis	13.09
Acid methyl ester	16.34	Cd2 ion	9.85	Pulverization	9.64	MoSe2	12.83
Sensitization	16.28	TiO2 nanotube array	9.22	Graphite anode	9.30	mAh g1	9.79
Solar energy conversion	14.13	Gold nanocage	8.76	Battery electrode	9.16	Visible light photocatalytic hydrogen	8.98
Catalytic growth	13.28	Nanometer pore diameter	8.76	Rechargeable lithium battery	8.53	Advanced electrode material	8.89
Butyric acid methyl ester	12.95	Silver nanocube	8.47	Charge capacity	8.13	Robust adhesion	8.42
Shell thickness	12.77	Polyaniline nanofiber	8.31	Energy storage device	7.48	Asymmetric supercapacitor	8.35
Core shell particle	9.93	Potentiostatic anodization	8.21	Cycle life	7.41	Flexible energy storage device	8.21
ZnO nanorod	9.83	Photoanode	7.42	Fascinating property	7.39	Superior cyclability	8.21
Electrospinning	9.55	Liberation	7.15	Supercapacitor electrode material	7.09	High energy lithium ion battery	8.11
Nanobelt	8.93	Titania nanotube array	6.80	High charge storage capacity	6.81	Capacitive energy storage	7.88
Silver nanowire	8.81	Block truncation coding	6.79	Electrochemical energy storage	6.80	New electrode material	7.87
Novel nanostructure	8.81	Chemical activity	6.46	Hydrocarbon fuel	6.77	Excellent mechanical strength	7.70
Soil	8.59	Lithium ion secondary battery	6.23	Capacity fading	6.75	High performance libs	7.66
Polyfluorene	8.42	Same chemical composition	6.16	Gradual reduction	6.64	Nanostructured electrode material	7.27

**Table 5:** Most relevant terms of Biotechnology and Biomedicine ﻿﻿﻿cluster﻿

2000–2003 (joined SNBUP)	Score	2004–2007	Score	2008–2010 (Biosensing)	Score	2008–2010 (Therapeutic biomedicine)	Score	2011–2013 (Biosensing)	Score	2011–2013 (Therapeutic biomedicine)	Score
ZnO nanowire	20.81	Photothermal therapy	9.97	Novel glucose biosensor	6.66	Abdominal cavity	8.63	Chemical class	10.07	Marine organism	6.91
Acid methyl ester	16.34	Unique optical property	8.85	Biotechnology application	6.32	Significant obstacle	7.28	Complex biological environment	5.82	Biological identity	6.00
Sensitization	16.28	Mesoporous silica nanoparticle	8.81	Droplet microfluidic	5.47	Harm	7.11	Luminogen	5.31	Cellular machinery	5.48
Solar energy conversion	14.13	Protein engineering	8.18	Excellent platform	5.25	Implantable medical device	6.23	Care diagnostic device	5.20	Tissue penetration depth	5.41
Catalytic growth	13.28	Nanometer nanoparticle	7.97	Graphene oxide surface	4.72	Surrogate	6.02	Existing camera unit	5.13	Unique size	5.22
Butyric acid methyl ester	12.95	Electrospun nanofiber	7.10	Norepinephrine	4.66	Vivo delivery	5.32	Dioxetane	5.04	Ce6	5.08
Shell thickness	12.77	Cellular toxicity	6.93	Complementary dna strand	4.55	Asbestos	5.25	Phosphoryloxy	5.04	Photodynamic therapy efficacy	4.72
Core shell particle	9.93	Arsenal	6.63	TNT detection	4.10	Apparent cytotoxicity	5.18	Powerful technology	4.26	Diseased cell	4.69
ZnO nanorod	9.83	S aureus	6.45	Bioelectronic	4.04	Novel form	5.15	Microfluidic paper	4.25	Modern medicine	4.65
Electrospinning	9.55	Gram-negative bacterium	6.42	Background fluorescence	4.03	Endosomal compartment	5.12	Resource limited setting	4.15	ICP MS analysis	4.48
Nanobelt	8.93	Cancer treatment	6.36	Non-enzymatic glucose sensor	4.01	Early onset	4.81	Aggregation-induced emission characteristics	4.07	High photothermal conversion efficiency	4.25
Silver nanowire	8.81	Cancer diagnosis	6.23	Wide linear response	3.89	Nanoparticle type	4.76	Microfluidic paper based analysis devices	4.06	New era	4.17
Novel nanostructure	8.81	Related technology	6.10	Epinephrine	3.82	Protein corona	4.66	Fluorogen	4.03	Controlled drug delivery	4.15
Soil	8.59	Antibacterial property	6.04	Novel graphene	3.78	Human exposure	4.48	Catecholamine	3.96	Vivo biocompatibility	4.08
Polyfluorene	8.42	Cancer diagnostic	5.85	Good electrocatalytic activity	3.75	Human cancer cell	4.41	Efficient platform	3.89	GelMA	4.07

**Table 6:** Most relevant terms of Microelectronics engineering and top-down processes cluster

2000–2003	Score	2004–2007	Score	2008–2010	Score	2011–2013	Score
Organic thin film transistor	11.50	Hydrophilic polymer	7.36	Few layer graphene films	16.71	Rich physic	16.04
Non-volatile memory	8.60	Novel nanoscale	7.17	Transparent conducting film	15.10	WSe2	12.19
Optical application	7.43	Superhydrophilicity	5.95	Exciting potential	13.48	PTB7	11.65
Design consideration	5.52	New architecture	5.66	Scalable technique	13.45	MoS2 monolayer	11.02
Ferroelectricity	5.36	Microscale device	5.36	Graphene electrode	12.58	Bilayer MoS2	10.10
Optical switch	4.95	Superhydrophobic coating	5.12	Nitrogen-doped graphene	12.14	Entire visible spectrum	9.65
Luminescence efficiency	4.20	High gain	4.94	Arbitrary substrates	11.20	Standard AM	9.47
Molecular monolayer	3.78	Superhydrophobicity	4.75	Large-scale growth	10.99	MoS2 transistor	9.08
Alkyl	3.71	Further optimization	4.57	Liquid phase exfoliation	10.80	Ionic motion	8.71
Energy consumption	3.61	Optical coherence tomography	4.19	Individual graphene sheet	10.62	Terminology	8.57
Carbon nanotube field effect transistor	3.44	Continuous flow separation	4.14	Large area graphene	10.18	Efficiency limitation	8.31
Strontium titanate	3.36	Enhanced mixing	4.06	Optoelectronic property	10.02	Valley polarization	8.14
Atomic force microscopy cantilever	3.34	Crust	4.03	Thick sheet	9.17	Efficient polymer solar cell	8.03
Conductive substrate	3.19	Bath temperature	3.99	Graphene oxide film	9.04	Reduced charge recombination	7.92
Charge fluctuation	2.97	Lithographic approach	3.85	Single sheet	8.93	PEC water splitting	7.80

**Table 7:** Most relevant terms of Physical and mechanical characteristics of materials cluster

2000–2003 (Mechanical)	Score	2000–2003 (Physical)	Score	2004–2007	Score	2008–2010	Score	2011–2013	Score
Solid oxide fuel cell	5.11	Magnesium alloy AZ31	5.66	Mechanical reinforcement	5.95	Enhanced strength	6.99	Local property	5.76
Stage process	3.66	Continuous dynamic recrystallization	3.96	Simultaneous increase	5.07	Effective reinforcement	3.74	Video recording	5.20
High capacity	3.54	Ultrafine grained	3.94	Annealing time t	3.24	Watt per meter Kelvin	2.92	Strong size dependence	3.73
Spark plasma	3.52	Accumulative roll bonding	3.08	Filler material	2.98	Dimensional finite element simulation	2.84	Tafel analysis	3.09
High temperature stability	3.24	Peak value	2.96	Nanotube polymer composite	2.84	Concrete	2.54	Random texture	2.57
Hall petch relationship	3.21	Nanocrystalline metal	2.69	MHz range	2.59	Ultimate strength	2.50	Si particle	2.45
Yttrium oxide	3.17	Recrystallization mechanism	2.46	Ultrafine grain size	2.54	Macromolecular structure	2.47	Relevant temperature range	2.31
Columnar grain structure	3.03	Flow instability	2.45	Low ductility	2.41	Disclination	2.18	Optimum balance	2.25
Coating property	3.02	Continuous recrystallization	2.43	Good electrical conductivity	2.40	Large strain	2.09	Primary focus	2.20
Coating stir weld	2.97	Route c	2.43	Ultrafine grained microstructure	2.38	Damaged region	2.09	Outstanding mechanical property	2.16
Solid matrix	2.97	High pressure torsion	2.39	Inducing	2.31	Minimally	2.07	Segregation behavior	2.16
Multicomponent system	2.92	Equal channel angular pressing	2.34	Surface mechanical attrition treatment	2.29	Major mechanism	2.04	Thick sheet	2.10
Fold symmetry	2.90	Silicon nanotube	2.31	Microwave sintering	2.25	Extrusion texture	2.02	Multistep	2.08
Load transfer	2.86	Hot deformation behavior	2.30	Deformability	2.20	Crystal form	1.86	LMC	1.99
Automotive industry	2.85	Grain size dependence	2.19	Rapid heating	2.08	Higher yield strength	1.86	Cyclic strain	1.96

**Table 8:** Most relevant terms of Organic electronics cluster

2008–2010	Score
Minority carrier diffusion length	11.72
Polymer donor	8.27
Vertical phase separation	8.19
High absorption coefficient	7.87
Fullerene acceptor	7.61
Polymer morphology	6.14
Solvent-free ionic liquid electrolyte	5.98
Reduced graphene oxide film	5.97
Photoconversion efficiency	5.76
Solution processed	5.75
Electron pathway	5.72
Functionalized graphene	5.52
Bulk heterojunction solar cell	5.49
Particulate film	5.46
Diketopyrrolopyrrole	5.46

*Optics and Electronics* (*yellow cluster*)

In most of the maps, this cluster occupied a central position, connected with the other clusters, a stem of sorts for the development of the NST network. Optics and Electronics provide foundations for chemical applications and for Biomedicine.

In the first period (2000–2003), *Optics and Electronics* appears closely linked in its upper sector to *Microelectronics engineering and top-down processes* and in its lower part to *Synthesis of nanomaterials and bottom-up processes.*

The following period (2004–2007) shows how this cluster begins to gain strength and influence in the rest of the research lines, with great relevance for *Synthesis of nanomaterials and bottom-up processes.*

From 2008 to 2010, *Optics and Electronics* is absorbed by the cluster *Synthesis of nanomaterials and bottom-up processes*, forming a single cluster. This union is due to the application of bottom-up processes and chemicals within *Optics and Electronics*, for instance optic sensors. At the end of the second study period, in 2007, the application of optic sensors takes off, out of the laboratory and into applied science. After 2008, *Optics and Electronics* is seen to approach the clusters *Synthesis of nanomaterials and bottom-up processes* and *Biotechnology and Biomedicine* (*Biosensing and Therapy*). In 2007, optic devices began to be used in biotechnology and medicine. At the same time, the nature of the material underwent development, first with inorganic and then with organic materials. For this reason, the term “quantum dot” disappears after 2008 and gives way to the entry of “graphene.”

The final period studied (2011–2013) continues to show a union of clusters *Optics and Electronics* and *Synthesis of nanomaterials and bottom-up processes* and suggests that these areas are bound to develop in tandem or even mutual dependence, eventually becoming one area.

*Synthesis of nanomaterials and bottom-up processes* (*green cluster*)

The cluster *Synthesis of nanomaterials and bottom-up processes* appears closely tied to the cluster *Optics and Electronics* because in NST, the bottom-up materials are widely studied for optical applications. These are in turn used in sensors for biodetection and for the treatment of disease (Biomedicine).

Like the cluster *Optics and Electronics*, this one first involves inorganic materials but then progresses toward the almost exclusive use of organic materials by 2009.

From 2000 to 2003, this cluster is fused to the cluster *Biotechnology and Biomedicine*. However, from 2004 to 2007, they separate, giving rise to two distinctive neighboring clusters. It was in this period when the nucleus of optic sensors underwent development. In the period of 2008–2010, this cluster appears linked to *Optics and Electronics*. Meanwhile, one sees that this cluster begins to interact with the cluster *Microelectronics engineering and top-down processes*, and we begin to see terms indicating the real applications of the top-down nanomaterials (microfluid, microchip, microchannel). At the same time, biosensors begin to be applied, namely, in 2008. In 2009, biosensors were introduced in microfluid devices and there was an explosion of output in biomedicine; hence, a new line of research appears. During the final interval of study (2011–2013), we find a new cluster next to this one, representing work with organic materials (*Organic electronics*).

*Biotechnology and Biomedicine* (*purple and orange clusters*)

The cluster *Biotechnology and Biomedicine* is linked to *Synthesis of nanomaterials and bottom-up processes* due to its use of this type of materials. It is also quite connected to *Optics and Electronics* because of optical measurements and to *Microelectronics engineering and top-down processes* because such devices are used as systems of detection.

In the first period of study, this cluster is joined to *Synthesis of nanomaterials and bottom-up processes*, but in the period of 2004–2007, the two clusters divide into separate research lines. Then, in the following period, given the scientific advances in *Optics and Electronics* and the *Synthesis of nanomaterials and bottom-up processes*, this cluster splits in two: *Biosensing* (purple cluster) and *Therapeutic biomedicine* (orange cluster)*.* In more recent years (2011–2013), *Biosensing* has captured more research, possibly because it provides more direct benefits for society.

*Microelectronics engineering and top-down processes* (*red cluster*).

The cluster *Microelectronics engineering and top-down processes* begins as a line of research related purely with electronics, but later, it begins to incorporate microfluids, thus shifting nearer to the cluster of *Biosensing*.

In 2000–2003, this cluster was only near *Optics and Electronics* (upper left). In the following period studied, the cluster grew substantially in size and part of its research came to border on *Biotechnology and Biomedicine* and *Physical and mechanical characteristics of materials*.

In the final period of study (2011–2013), the left part of the cluster *Microelectronics engineering and top-down processes* approaches *Biosensing*, as the terms represented here frequently refer to microfluids, commonly used for biodetection. At the same time, this cluster becomes less compact, opening toward the right until it is situated parallel to the cluster *Synthesis of nanomaterials and bottom-up processes*, thereby demonstrating its influence in the rest of the disciplines within NST.

*Physical and mechanical characteristics of materials* (*blue and pink clusters*)

The cluster *Physical and mechanical characteristics of materials* could be considered the first research line to be developed in the field of NST. The studies along this line are related with the principals of physics and the study of the mechanical properties or physical characterizations of certain materials in conjunction with NST discoveries. Once these materials were more familiar at the nanometric scale, the line of research slowed down, while other lines began to evolve thanks to the discoveries recently made. Hence, the lines are seen to intersect or interact to some degree. The tendency over the years is for the cluster *Physical and mechanical characteristics of materials* to move away from the rest, although it still shares limited activity with *Optics and Electronics*, *Synthesis of nanomaterials and bottom-up processes*, and *Microelectronics engineering and top-down processes*. The interaction can be traced to the employment of materials common to all the clusters, yet the part dedicated to the study of mechanical properties of materials tends toward isolation.

During the period of 2000–2003, this cluster lies at a great distance from the other research lines. Another singular aspect of this cluster is its division into two lines. Namely, *Physical characteristics of materials* (pink cluster, top left) and *Mechanical characteristics of materials* (blue cluster, lower left). We also see a distance from the other research lines identified. Yet, in the following period (2004–2007), the two lines fuse into a single cluster and remain attached until the end of the period.

*Organic electronics (light blue cluster).*

This cluster only appears in the period of 2008–2010, owing to the use of organic materials within the realm of NST research. This causes it to occupy a position nearby the cluster *Synthesis of nanomaterials and bottom-up processes* However, the line of research eventually disappears, absorbed into the cluster *Microelectronic engineering and top-down processes* during 2011–2013.

## Conclusions

By means of CitnetExplorer, we have analyzed the intellectual structure of nanoscience and nanotechnology, identifying the seminal papers and key documents of all NST journals contained in the JCR Science Edition during the period 2000–2013.

In view of the influence that the most-cited works have exerted over the years studied, we conclude that seven groups can be discerned within the intellectual structure of NST. The articles making up these groupings are essential papers of reference for the development of the discipline, and they are recognized as such through citations by the community of scientists. The underlying structure visualized here shows that even a limited and recent time period serves to elaborate the genealogy of a field such as NST, as the scientific culture always leaves footprints behind—that is, new authors look to their predecessors for foundational knowledge (Bayer et al. 1990). Citation habits are strongly conditioned by the magnitude of scientific progress, as reflected by the articles by Iijima about carbon nanotubes, or the experiments of Novoselov and colleagues with graphene. Such crucial steps in the development of NST give rise to new subject areas that are eventually consolidated through an array of articles, either within or surrounding the intellectual sphere of *Carbon nanomaterials*.

Deserving mention as an outstanding example is the appearance of a thematic area related with *Organic electronics*, in this case attracting little attention among the scientific community, with only one article in the map cluster.

In our opinion, the structures identified and displayed in this work make manifest certain consolidated subject areas, stemming from works that report authentic scientific breakthroughs or key methodological proposals. There is evident consensus regarding the significance and usefulness of such contributions to NST.

Many of the documents identified in this study differ from those described by Kostoff et al. (2006a, 2007a), who took into account the citation of the documents of all scientific fields. Our work resorted to citation of the documents in the category NST of the WoS, meaning that we only accounted for citation by experts in the discipline. Visualization techniques and co-word analysis, in this case from titles and abstracts, allowed us to identify and project the evolution of the main research lines developed in NST from 2000 up to 2013.

The research lines traced are evidence of output exploring the behavior and phenomena at the nanoscale, together with the creation of new materials and their diverse applications. The profile of NST, accordingly, can be summed up as a young discipline in steady expansion, still needing some time to secure its foundations. This would explain why research lines such as *Optics and Electronics*, *Synthesis of nanomaterials and bottom-up processes*, or *Physical and mechanical characteristics of materials* maintain a constant flow of output. In contrast, *Biotechnology and Biomedicine* and *Microelectronics engineering and top-down processes* undergo a bit of a boom, perhaps because there are greater health-related social benefits associated with these topics. It could also be that these lines will produce important economic benefits in the near future. New research lines arise with the applications of nanomaterials together with the trend of working with organic materials, as seen in the case of *Organic electronics*. However, no research line related with NST toxicology in biological or environmental contexts was detected. Many words related to this field— nanoparticle toxicity, nanotoxicity, ecotoxicity, and so on—are included in the clusters *Biotechnology and Biomedicine: Biosensing and Therapeutic biomedicine.* This line will no doubt emerge and gain strength in the coming years, with new developments to generate methods for the safe production, use, and elimination of nanomaterials (Nel et al. 2006).

When the number of documents published in a given research area is lower than in others, the number of citation is understandably low as well. Here, we could evoke the case of nanotoxicity. While some National Science Foundation (NSF) funded centers investigate the toxicity of nanomaterials (e.g., the Center for the Environmental Implication of Nanotechnology, Center for Sustainable Nanotechnology), overall, the production of scientific literature in this area that does not strictly correspond to *Biotechnology and Biomedicine* or *Synthesis of nanomaterials and bottom-up processes* may be low because its scope of action is limited. It may also be that scientific production in this specific realm is not registered in the leading multidisciplinary database of WoS. We might therefore affirm that our work has not yet come to an end along this research line: one of the journals that our search strategy includes is Nanotoxicology, a specialized journal in this field. On the other hand, one journal is not enough to provide insightful clues into a field of knowledge. On a broader panorama, as the science maps show, all the terms associated with nanotoxicity appear inside the clusters *Biotechnology and Biomedicine* or *Synthesis of nanomaterials and bottom-up processes*, not as a separate research line.

The lack of scientific literature in the wake of certain research lines and the low citation rate harvested by them could explain why most seminal papers identified in this manuscript pertain to the field of technology development.

Both the intellectual and the cognitive structures identified illustrate how NST continues to occupy a largely interdisciplinary scientific area. Indeed, its development depends on the knowledge that comes from physics, chemistry, and materials science, as displayed by the science maps. This is no novelty, having been endorsed by previous analyses (Meyer and Persson 1998; Schummer 2004; Porter and Youtie 2009; Jo et al. 2016), which to some extent demonstrate the convergence and validity of the scientometric methodology used in this study. In short, however, the present endeavor strives to trace the research lines of NST and thereby show how research in the area has changed over time. It is our hope that this study will become a useful tool of reference for the NST scientific community, revealing at a glance essential research lines in the light of landmark papers. Additionally, the methodology used in this study can be implemented in any other scientific field so as to explore its intellectual and cognitive structure.

### Limitations and future efforts

We must acknowledge specific problems inherent to map elaboration. Firstly, when working with a free and non-controlled language for the titles and abstracts of the documents studied, there are inevitable problems of synonymy, acronymy, and plural vs. singular denoting a single concept. Secondly, the non-use of controlled language may have led to an absence of keywords that could define a research line quite well but were not included in the titles or abstracts that we worked with. A potential solution for the future is to use keyword authors and index keywords to create science maps. This would necessarily entail using both controlled and non-controlled language, because the use of controlled language alone implies an absence of terms presently used in NST (Braam et al. 1991b).

Another limitation is related to the specific knowledge of the studied subject (NST) that will be covered in future works. No doubt consultation with experts in the scientific field would solve this limitation, since the opinion, review, and comments from them would allow us to guarantee the results, as well as reinforce, reorganize, reclassify, and deepen the thematic specialization, and therefore delimit in a clearer and more concise way the groups pertaining to the network and co-word maps.

Looking once again toward the future, we believe that the design of search strategies will be improved, embracing journals that cover all the research lines that NST touches upon and not merely the journal set ascribed under WoS. Furthermore, the methodology put forth here may be exported to study the category NST in other databases of a multidisciplinary character, where this category has been previously identified. It could also be extrapolated to other thematic fields. In addition, overlay techniques may be used in conjunction with the method. To a certain extent, the future of bibliometric techniques for scientific categorization and exploration may be foreseen through similar efforts.

## Appendix A. Abbreviated journal titles (JCR) query

SO = (ACM J EMERG TECH COM OR ACS APPL MATER INTER OR ACS NANO OR ADV FUNCT MATER OR ADV MATER OR AIP ADV OR BEILSTEIN J NANOTECH OR BIOMED MICRODEVICES OR BIOMICROFLUIDICS OR BIOSENS BIOELECTRON OR CURR NANOSCI OR DIG J NANOMATER BIOS OR FULLER NANOTUB CAR N OR IEEE T NANOBIOSCI OR IEEE T NANOTECHNOL OR IET NANOBIOTECHNOL OR INT J NANOMED OR INT J NANOTECHNOL OR J BIOMED NANOTECHNOL OR J COMPUT THEOR NANOS OR J EXP NANOSCI OR J LASER MICRO NANOEN OR J MICRO-NANOLITH MEM OR J MICROMECH MICROENG OR J NANO RES-SW OR J NANOBIOTECHNOL OR J NANOELECTRON OPTOE OR J NANOMATER OR J NANOPART RES OR J NANOPHOTONICS OR J NANOSCI NANOTECHNO OR J PHYS CHEM C OR J PHYS CHEM LETT OR J VAC SCI TECHNOL B OR LAB CHIP OR MAT SCI ENG A-STRUCT OR MATER EXPRESS OR MICRO NANO LETT OR MICROELECTRON ENG OR MICROELECTRON J OR MICROELECTRON RELIAB OR MICROFLUID NANOFLUID OR MICROMACHINES-BASEL OR MICROPOR MESOPOR MAT OR MICROSYST TECHNOL OR NANO OR NANO ENERGY OR NANO LETT OR NANO RES OR NANO TODAY OR NANO-MICRO LETT OR NANOMATER NANOTECHNO OR NANOMED-NANOTECHNOL OR NANOMEDICINE-UK OR NANOSC MICROSC THERM OR NANOSCALE OR NANOSCALE RES LETT OR NANOSCI NANOTECH LET OR NANOTECHNOLOGY OR NANOTOXICOLOGY OR NAT NANOTECHNOL OR PART PART SYST CHAR OR PHOTONIC NANOSTRUCT OR PHYSICA E OR PLASMONICS OR PRECIS ENG OR RECENT PAT NANOTECH OR REV ADV MATER SCI OR SCI ADV MATER OR SCRIPTA MATER OR SMALL OR SYNTH REACT INORG M OR WIRES NANOMED NANOBI)

## Appendix B. Nodes and citations

**Table:**

Node	Year	Source	Citation
Iijima	1991	Nature	3183
Perdew	1996	Physical Review Letters	3072
Novoselov	2004	Science	2856
Geim	2007	Nature Materials	2169
Kresse	1996	Physical Review B	1997
Oregan	1991	Nature	1779
Xia	2003	Advanced Materials	1531
Kresse	1999	Physical Review B	1529
Frisch	2004	Gaussian 03 Revision	1492
Kresse	1996	Computational Materials Science	1462
Huang	2001	Science	1444
Monkhorst	1976	Physical Review B	1441
Blochl	1994	Physical Review B	1438
Kresge	1992	Nature	1343
Alivisatos	1996	Science	1311
Daniel	2004	Chemical Reviews	1258
Novoselov	2005	Nature	1201
Baughman	2002	Science	1197
Lee	1988	Physical Review B	1196
Becke	1993	Journal of Chemical Physics	1172
Hummers	1958	Journal of the American Chemical Society	1135
Murray	1993	Journal of the American Chemical Society	1077
Johnson	1972	Physical Review B	1075
Cui	2001	Science	1063
Pan	2001	Science	1041
Wagner	1964	Applied Physics Letters	1029
Nie	1997	Science	1029
Kresse	1993	Physical Review B	1026
Zhang	2005	Nature	1026
Sze	1981	Phys Semiconductor Devices	1010
Chan	1998	Science	1001
Bruchez	1998	Science	996
Kelly	2003	Journal of Physical Chemistry B	992
Zhao	1998	Science	951
Law	2005	Nature Materials	950
Stankovich	2006	Nature	918
Fujishima	1972	Nature	913
Kim	2009	Nature	903
Gratzel	2001	Nature	890
Li	2009	Science	889
Michalet	2005	Science	876
Ferrari	2006	Physical Review Letters	875
Kong	2000	Science	870
Perdew	1992	Physical Review B	861
Hoffmann	1995	Chemical Reviews	845
Vanderbilt	1990	Physical Review B	844
Burda	2005	Chemical Reviews	841
Beck	1992	Journal of the American Chemical Society	827
Lee	2008	Science	825
Perdew	1992	Physical Review B	818
Decher	1997	Science	799
Tans	1998	Nature	786
Castro Neto	2009	Reviews of Modern Physics	786
Morales	1998	Science	780
Huynh	2002	Science	777
Saito	1998	Phys Properties Carb	759
Geim	2009	Science	750
Kohn	1965	Physical Review	736
Wang	2006	Science	724
Li	2008	Nature Nanotechnology	724
Stankovich	2007	Carbon	719
Kreibig	1995	Optical Properties M	713
Frisch	2009	Gaussian 09 Revision	708
Love	2005	Chemical Reviews	702
Asahi	2001	Science	700
Barnes	2003	Nature	697
Peng	2000	Nature	690
Soler	2002	Journal of Physics: Condensed Matter	681
Medintz	2005	Nature materials	672
Novoselov	2005	PNAS	659
Schedin	2007	Nature Materials	634
Yu	2003	Chemistry of Materials	633
Bard	2001	Electrochemical Meth	632
Berger	2006	Science	629
Kresse	1994	Physical Review B	628
Troullier	1991	Physical Review B	621
Bohren	1983	Absorption Scattering	619
Li	2008	Science	616
Li	2005	Nature Materials	614
Kneipp	1997	Physical Review Letters	607

## Footnotes

1 This thesaurus was generated by a text editor, and then imported to VOSviewer. It contains approximately 2000 equivalencies and is available at *http://www.ugr.es/local/benjamin/thesaurus-N&N.txt*.
